# Baseline peak expiratory flow and incident hip fracture in older Chinese adults: A prospective CHARLS cohort study

**DOI:** 10.1097/MD.0000000000051167

**Published:** 2026-10-23

**Authors:** Wei Chen, Zongfeng Chen, Yan Wang, Wanfei Wu, Yufeng Zhao, Jun Fei

**Affiliations:** a War Trauma Medical Center, Army Medical Center, Daping Hospital, Army Medical University, Chongqing, PR China.

**Keywords:** CHARLS, cohort study, geriatrics, hip fracture, peak expiratory flow, pulmonary function

## Abstract

Peak expiratory flow (PEF), a simple measure of respiratory function, may be linked to skeletal health through shared pathways related to physiological reserve and functional capacity. However, the long-term association between PEF and incident hip fracture in community-dwelling older adults remains unclear. We conducted a longitudinal analysis of participants aged ≥60 years from the China Health and Retirement Longitudinal Study, using 2013 as baseline and follow-up waves in 2015, 2018, and 2020 (up to ~7 years). Baseline PEF was defined as the maximum of 3 attempts, and incident hip fracture was identified as self-reported hip fracture first reported during follow-up. Associations were evaluated using Cox proportional hazards models stratified by self-rated health, with multiple imputation for missing covariates. PEF was modeled per 1-standard deviation increase and by quartiles. A prespecified conceptual framework distinguished likely confounders from potentially pathway-related variables. Supplementary discrimination analyses used C-index and time-dependent area under the receiver operating characteristic curve. Among 5789 participants, incident hip fractures occurred in 98 (6.5%), 76 (5.1%), 51 (3.7%), and 46 (3.2%) individuals from the lowest to highest PEF quartile (*P* < .001). Per 1-standard deviation higher PEF, hazard ratios were 0.70 (95% CI: 0.62–0.80) in the crude model, 0.79 (0.68–0.91) in the primary confounder-adjusted model, and 0.87 (0.74–1.02) in the broader model. For quartiles, Q4 versus Q1 hazard ratios were 0.46 (0.32–0.65), 0.63 (0.43–0.93), and 0.80 (0.53–1.20), respectively. Restricted cubic splines showed no evidence of nonlinearity (*P* = .866). Supplementary discrimination analyses showed higher C-index and time-dependent area under the receiver operating characteristic curves for the broader model than for the unadjusted and primary confounder-adjusted models. Higher baseline PEF was associated with lower incident hip fracture risk in crude and partially adjusted analyses, but the association was attenuated after broader adjustment. Baseline PEF appears to have limited stand-alone prognostic value and may be better viewed as a pragmatic signal prompting broader geriatric assessment rather than as a stand-alone fracture risk tool.

## 1. Introduction

Hip fracture remains one of the most consequential injuries in later life, often leading to substantial functional decline, loss of independence, and excess mortality. Global analyses indicate that the absolute number of fractures has increased markedly over recent decades, with the highest incidence observed in the oldest age groups.^[1]^ Despite advances in acute management, hip fracture continues to be associated with high short-term mortality, with considerable variation across settings and persistent gaps in secondary prevention.^[2]^ In clinical practice, widely used fracture risk assessment tools rely largely on bone mineral density and traditional clinical risk factors and may not fully capture multisystem vulnerability – such as frailty, sarcopenia, and diminished physiologic reserve – that contributes to falls and hip fracture risk.

In patients with hip fracture, perioperative pulmonary vulnerability is clinically relevant because postoperative complications such as pneumonia and respiratory failure contribute to prolonged hospitalization and greater monitoring needs, including ICU-level care in some cases.^[3-5]^ This clinical context raises an upstream question: whether poorer respiratory function in the community is associated with subsequent hip fracture, potentially as a marker of broader multisystem vulnerability rather than a fracture-specific mechanism. Because full spirometry is not always feasible in large-scale surveys or routine geriatric assessment, peak expiratory flow (PEF), a simple and low-cost bedside measure, may be useful as a pragmatic indicator of reduced physiologic reserve and a signal for broader geriatric risk assessment.

PEF is a simple and low-cost measure of expiratory function that can be obtained in community-based surveys and routine clinical care. In older adults, PEF reflects not only airway caliber but also respiratory muscle performance and overall ventilatory capacity, and it has been proposed as a pragmatic indicator of respiratory impairment when spirometry is unavailable or not feasible.^[6]^ Impaired respiratory function may plausibly relate to hip fracture risk through shared pathways, including systemic inflammation, reduced physical capacity, sarcopenia, and frailty – factors that increase fall susceptibility and may adversely affect bone–muscle health.^[7-11]^ Consistent with this conceptual framework, observational evidence indicates that lower spirometric function is associated with increased risk of osteoporosis and hip fracture.^[12]^ Moreover, low PEF has been associated with a higher risk of injurious falls in older adults, supporting a potential link between respiratory impairment, reduced physical function, and fracture-related outcomes.^[13]^ In addition, chronic respiratory disease such as chronic obstructive pulmonary disease has been reported to increase hip fracture risk, further suggesting a respiratory–skeletal vulnerability axis.^[14]^

However, epidemiologic evidence specifically linking PEF to incident hip fracture remains limited. Existing studies have more often examined spirometric indices such as forced expiratory volume in 1 second (FEV1) or broader respiratory disease–related measures, and several influential cohorts were conducted in Western populations, which may limit direct generalizability to Asian settings with different body composition, risk profiles, and healthcare contexts.^[12,14,15]^ In addition, relatively few longitudinal analyses have comprehensively evaluated key issues relevant to clinical interpretation, including time-to-event modeling across multiple follow-up waves, assessment and accommodation of proportional hazards assumptions, characterization of dose–response relationships, and quantification of discriminatory performance over time.

To address these gaps, we used data from the China Health and Retirement Longitudinal Study (CHARLS), a nationally representative longitudinal cohort with standardized interviews and physical measurements, to examine the prospective association between baseline PEF and incident hip fracture among older Chinese adults.^[16]^ We followed participants across 3 subsequent waves (2015/2018/2020; up to ~7 years) and evaluated associations using multivariable Cox models, complemented by Kaplan–Meier analyses, prespecified subgroup and interaction assessments, restricted cubic splines (RCS) to characterize the dose–response pattern. Together, these analyses aimed to clarify whether a readily obtainable respiratory measure is associated with incident hip fracture and how this association changes after adjustment for prespecified confounding and pathway-related domains in an aging population.

## 2. Materials and methods

### 2.1. Study design and data source

This was a prospective cohort analysis of a nationally representative community-based cohort derived from the CHARLS. CHARLS is an ongoing survey of Chinese adults with standardized interviews and physical measurements. The 2013 survey (wave 2) was used as the baseline, and participants were followed in 2015 (wave 3), 2018 (wave 4), and 2020 (wave 5), providing approximately 7 years of follow-up. Hip-fracture time-to-event was constructed using the first follow-up wave in which a hip fracture was reported; thus, event times were discrete and typically corresponded to ~2, ~5, or ~7 years from baseline. The CHARLS protocol was approved by the Ethical Review Committee of Peking University (IRB00001052-11015), and all participants provided written informed consent at enrollment.

### 2.2. Study participants

Participants were eligible if they: were aged ≥60 years at baseline (2013), had a valid baseline PEF measurement, reported no history of hip fracture at baseline, and (4) had hip-fracture outcome information available in at least 1 follow-up wave (2015–2020). Participants were excluded if they were <60 years, had missing baseline PEF, reported a baseline hip fracture, or had missing hip-fracture outcome information across all follow-up waves. The final analytic sample comprised 5789 participants (participant flow shown in Fig. 1). Participants without a hip fracture were censored at the last wave with available hip-fracture information.

**Figure 1.**
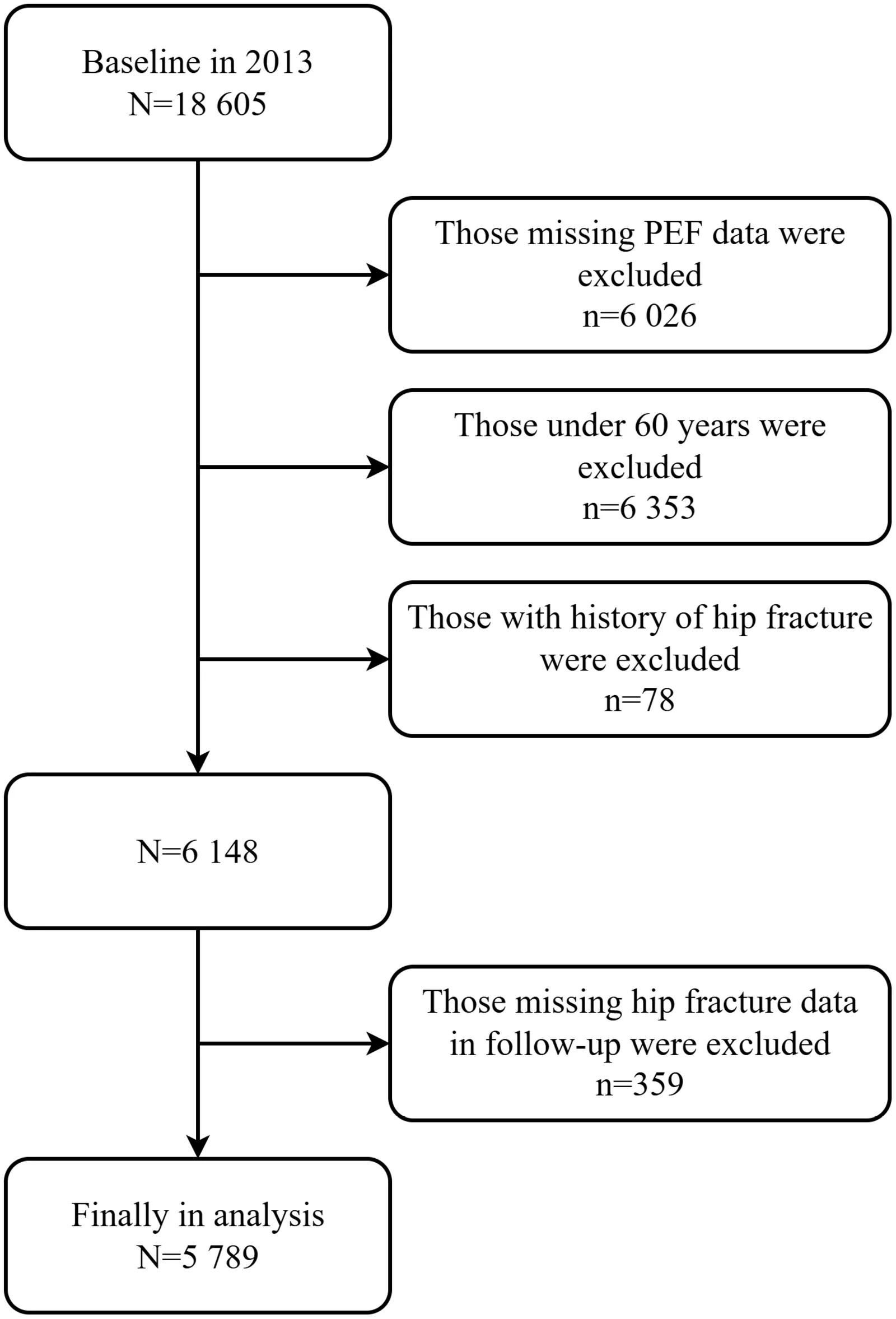
Flowchart of participant selection and derivation of the analytic cohort. PEF = peak expiratory flow.

### 2.3. Measurement of exposure and outcome

#### 2.3.1. Exposure (peak expiratory flow)

Baseline PEF (L/min) was measured using a peak flow meter with a disposable mouthpiece. Participants were instructed to stand, inhale deeply, seal their lips around the mouthpiece, and exhale “as hard and as fast as possible.” Three attempts were recorded. Values coded as “refused” were treated as missing. Values coded as “unable to complete” were recoded to a predefined minimum value per the CHARLS coding scheme. The final PEF exposure was defined as the maximum of the 3 measurements, provided not all 3 were missing. Because PEF is effort-dependent, the baseline measure should be interpreted as a pragmatic field measure rather than a formal spirometric parameter.^[17,18]^

PEF was analyzed as a continuous variable (per 1 standard deviation [SD] increase) and in quartiles (Q1–Q4). To ensure identical exposure coding across multiply imputed datasets, the PEF mean/SD (for *z*-scoring) and quartile cut-points were derived from the first completed dataset from the imputed object and then applied unchanged to all imputations; Q1 (lowest quartile) was the reference group.

#### 2.3.2. Outcome (incident hip fracture)

The outcome was incident hip fracture during follow-up, defined as self-reported hip fracture first reported in any follow-up wave (2015/2018/2020). The event time was assigned to the first wave reporting hip fracture; participants without a reported hip fracture were censored at their last wave with outcome information. Because the outcome was self-reported rather than adjudicated by imaging or medical records, some outcome misclassification is possible.^[19,20]^

### 2.4. Assessment of covariates

Potential confounders were prespecified based on clinical relevance and prior literature. Baseline covariates included age, sex, body mass index (BMI), hypertension, diabetes, cancer, heart disease, lung disease, stroke, arthritis, physical disabilities, smoking status, alcohol consumption, hospitalization in the past year, number of falls in the past year, activities of daily living (ADL), instrumental activities of daily living (IADL), cognitive function, depressive symptoms, education level, hukou type, marital status, and self-rated health.

#### 2.4.1. ADL/IADL scoring

ADL was assessed using 6 items (dressing, bathing/showering, eating, getting into/out of bed, using the toilet, and controlling urination/defecation) with 4 ordered response categories. Each item was dichotomized as independent (“no difficulty” or “difficulty but still able to do,” score = 1) vs dependent (“need help” or “unable to do,” score = 0). The ADL total was summed across items (range 0–6). IADL was assessed using 6 items (housekeeping, preparing hot meals, grocery shopping, making phone calls, managing money, taking medications) and dichotomized analogously. For the phone-call item, “not relevant” was treated as missing. ADL and IADL totals were calculated only when ≥4 of 6 items had valid responses; otherwise, totals were set to missing.

A prespecified conceptual framework was developed to distinguish baseline variables more likely to act as confounders from those potentially lying along the pathway between lower PEF and incident hip fracture (Fig. S1, Supplemental Digital Content 1). In this framework, demographic, socioeconomic, lifestyle, and baseline comorbidity variables were considered likely confounders, whereas functional status, cognitive/depressive status, and falls were considered potentially pathway-related variables. Self-rated health was treated separately because it was used as a stratification factor in the Cox models.

### 2.5. Statistical analysis

#### 2.5.1. Descriptive analysis

Baseline characteristics were summarized across PEF quartiles and compared across quartiles; corresponding *P* values were reported.

#### 2.5.2. Proportional hazards assessment

Proportional hazards assumptions were assessed using Schoenfeld residual-based diagnostics. Self-rated health was dichotomized as satisfied versus not satisfied and was used as a stratification factor because proportional hazards diagnostics suggested nonproportionality.

#### 2.5.3. Primary time-to-event analysis

Associations between baseline PEF and incident hip fracture were evaluated using Cox proportional hazards regression with Efron’s method for ties. Three models were fitted: model 1 (crude), model 2 (primary confounder-adjusted; adjusted for age, sex, hukou type, education level, and marital status), and model 3 (broader conservative/exploratory model; additionally adjusted for IADL, ADL, cognitive score, 10-item Center for Epidemiologic Studies Depression Scale, BMI, hypertension, diabetes, cancer, heart disease, lung disease, stroke, arthritis, physical disabilities, smoking, drinking, hospitalization in the past year, and falls in the past year, and stratified by self-rated health). Model 2 was treated as the primary confounder-adjusted model. Model 3 was not interpreted as a strict causal model; rather, it was used to assess attenuation of the association after broader adjustment that included variables plausibly related to functional vulnerability and potential pathway-related domains. Results are presented as hazard ratios (HRs) with 95% confidence intervals (CIs). *P* values for linear trend across quartiles were obtained by modeling quartile medians as an ordinal term. Pathway-adjusted models were descriptive and were not interpreted as formal mediation analyses.

#### 2.5.4. Kaplan–Meier analysis

Hip fracture-free survival was visualized using Kaplan–Meier curves across baseline PEF quartiles, and differences were evaluated using the log-rank test.

#### 2.5.5. Dose–response analysis

RCS were used to evaluate potential nonlinearity between continuous PEF and hip fracture risk, using 4 knots. The spline model was fitted within the Cox framework with stratification by self-rated health. Nonlinearity was tested by the significance of the nonlinear spline component (analysis of variance). Spline-based HR curves were plotted with the reference value set to the median PEF.

#### 2.5.6. Subgroup and interaction analyses

Prespecified subgroup analyses estimated HRs for PEF (per 1-SD increase) within strata of demographic, lifestyle, functional, and comorbidity variables. Interaction was tested by comparing models with and without a PEF × subgroup interaction term.

#### 2.5.7. Sensitivity analyses

Given the discrete nature of outcome ascertainment across waves, sensitivity analyses used a discrete-time hazard approach by constructing a person-period dataset and fitting complementary log–log models to approximate grouped-time proportional hazards. Additional sensitivity analyses excluded participants with early hip fracture by the 2015 wave and excluded those who died during follow-up. Sensitivity models were pooled across imputations. Death was considered an important competing event; however, formal competing-risk regression was not feasible because the analytic dataset only identified whether death occurred during follow-up by the final wave, whereas sufficiently precise time-to-death information was available for only a small minority of deaths.

#### 2.5.8. Supplementary discrimination analyses

To provide supplementary information on model discrimination, we calculated Harrell’s C-index and time-dependent area under the receiver operating characteristic curve (AUC) at 2, 5, and 7 years for model 1, model 2, and model 3 within each imputed dataset and summarized these measures across imputations (Table S2, Supplemental Digital Content 2). These analyses were intended as supplementary discrimination analyses rather than formal predictive model development or external validation.

#### 2.5.9. Missing data and multiple imputation

Missing covariate data (Table S1, Supplemental Digital Content 3) were imputed using multiple imputation by chained equations implemented in the mice package in R (version 4.4.3; R Foundation for Statistical Computing). We generated 20 imputed datasets with 20 iterations each. Continuous variables were imputed using predictive mean matching; binary variables using logistic regression; nominal categorical variables using polytomous regression; and ordinal variables using proportional odds models. The outcome-related variables were not imputed; outcome variables were included as predictors in the imputation models. All analyses were performed within each imputed dataset and pooled using Rubin’s rules. For supplementary discrimination analyses, measures were calculated within each imputed dataset and summarized across imputations.

#### 2.5.10. Software and significance

All analyses were performed in R (version 4.4.3). Two-sided *P* values < .05 were considered statistically significant.

## 3. Results

### 3.1. Study population

Of 18,605 baseline respondents, we excluded those with missing baseline PEF (n = 6026), those aged <60 years (n = 6353), those reporting a prior hip fracture at baseline (n = 78), and those missing hip-fracture outcome information in all follow-up waves (n = 359). The final analytic cohort included 5789 participants (Fig. 1).

### 3.2. Baseline characteristics

Baseline characteristics by PEF quartiles (Q1, 30–170 L/min; Q2, >170–250 L/min; Q3, >250–340 L/min; and Q4, >340–890 L/min) are presented in Table 1. Participants in the lowest PEF quartile (Q1) were older (mean age 70.8 ± 7.5 years) and had a lower proportion of males (38.1%) compared with those in the highest quartile (Q4; mean age 65.6 ± 5.0 years; 79.9% male). Across increasing PEF quartiles, BMI increased (Q4: 24.0 ± 3.5 kg/m^2^), and measures of cognition and depressive symptoms showed more favorable distributions. Lung disease, arthritis, and stroke were more prevalent in lower PEF quartiles. During follow-up, incident hip fractures occurred in 98 (6.5%), 76 (5.1%), 51 (3.7%), and 46 (3.2%) participants from Q1 to Q4, respectively, indicating a decreasing gradient across quartiles (*P* < .001; Table 1).

**Table 1:** Baseline characteristics of participants.

Characteristics	PEF quartiles				
	Q1 (n = 1499)	Q2 (n = 1489)	Q3 (n = 1361)	Q4 (n = 1440)	*P* value
Demographic					
Age, yr	70.8 ± 7.5	68.2 ± 6.2	67.0 ± 6.0	65.6 ± 5.0	*<*.001
Male, n (%)	571 (38.1)	552 (37.1)	607 (44.6)	1151 (79.9)	*<*.001
Married, n (%)	1026 (68)	1129 (76)	1086 (80)	1250 (87)	*<*.001
Hukou type					*<*.001
Agriculture	1258 (84)	1208 (81)	1066 (78)	992 (69)	
Non-agriculture	230 (15)	265 (18)	283 (21)	430 (30)	
Other	11 (0.7)	16 (1.1)	12 (0.9)	18 (1.3)	
Smoking status					*<*.001
No smoking	990 (66)	1057 (71)	944 (69)	752 (52)	
Quit	188 (13)	144 (9.7)	153 (11)	275 (19)	
Still smoking	321 (21)	288 (19)	264 (19)	413 (29)	
Drinking status					*<*.001
More than once a month	1128 (75)	1094 (73)	951 (70)	734 (51)	
Less than once a month	91 (6.1)	92 (6.2)	96 (7.1)	135 (9.4)	
None of these	280 (19)	303 (20)	314 (23)	571 (40)	
Education level					*<*.001
Primary school or below	1036 (69)	930 (62)	754 (55)	541 (38)	
Secondary school	409 (27)	506 (34)	515 (38)	730 (51)	
Above secondary school	54 (3.6)	53 (3.6)	92 (6.8)	169 (12)	
Physical and functional					
BMI, kg/m^2^	22.4 ± 3.8	23.3 ± 3.6	23.8 ± 3.7	24.0 ± 3.5	*<*.001
IADL	6 [5–6]	6 [5–6]	6 [5–6]	6 [6–6]	*<*.001
ADL	6 [6–6]	6 [6–6]	6 [6–6]	6 [6–6]	*<*.001
Cognitive score	7 [4–11]	8 [5–12]	10 [6–13]	12 [9–14]	*<*.001
CESD-10	8 [5–13]	7 [4–12]	7 [4–11]	6 [3–9]	*<*.001
Self-rated health (satisfied), n (%)	591 (39)	663 (45)	651 (48)	775 (54)	*<*.001
Health status					
Hospitalization past year, n	0 [0–0]	0 [0–0]	0 [0–0]	0 [0–0]	*<*.001
Number of falls, n	0 [0–0]	0 [0–0]	0 [0–0]	0 [0–0]	.004
Hypertension, n (%)	436 (29.1)	419 (28.1)	396 (29.1)	391 (27.2)	.611
Diabetes, n (%)	90 (6.0)	79 (5.3)	99 (7.3)	110 (7.6)	.038
Cancer, n (%)	13 (0.9)	15 (1.0)	12 (0.9)	8 (0.6)	.590
Heart disease, n (%)	219 (14.6)	220 (14.8)	190 (14.0)	185 (12.8)	.429
Lung disease, n (%)	326 (21.7)	168 (11.3)	110 (8.1)	90 (6.2)	*<*.001
Stroke, n (%)	47 (3.1)	51 (3.4)	33 (2.4)	27 (1.9)	.048
Arthritis, n (%)	573 (38.2)	590 (39.6)	482 (35.4)	451 (31.3)	*<*.001
Physical disabilities, n (%)	52 (3.5)	45 (3.0)	37 (2.7)	39 (2.7)	.571
Hip fracture, n (%)	98 (6.5)	76 (5.1)	51 (3.7)	46 (3.2)	*<*.001

Continuous variables with normal distribution are presented as mean ± standard deviation, while those with non-normal distribution as median [interquartile range]; categorical variables as n (%).

*P* values were calculated using pooled D1 statistics (multivariate Wald tests) based on 20 multiply imputed datasets. Linear regression models were used for continuous variables, logistic regression for binary clinical characteristics, and proportional odds models for sociodemographic and lifestyle variables (treating PEF quartiles as the ordinal outcome).

Statistical significance was set at *P* < .05.

ADL = activities of daily living, BMI = body mass index, CESD-10 = 10-item Center for Epidemiologic Studies Depression Scale, IADL = instrumental activities of daily living, PEF = peak expiratory flow.

### 3.3. Primary analysis

Proportional hazards diagnostics indicated potential nonproportionality for self-rated health; therefore, self-rated health was included as a stratification factor in the final Cox models. In Cox regression (Table 2), higher baseline PEF was associated with lower incident hip fracture risk in model 1 and model 2. Per 1-SD increase in PEF, the HR was 0.70 (95% CI: 0.62–0.80) in the crude model and 0.79 (95% CI: 0.68–0.91) after adjustment for age, sex, hukou type, education level, and marital status. In the broader, conservative/exploratory model (model 3), the association was attenuated and was not statistically significant (HR 0.87, 95% CI: 0.74–1.02).

**Table 2:** Association between peak expiratory flow and hip fracture risk.

Variable	Hazard ratio (95% CI)		
	Model 1*	Model 2†	Model 3‡
PEF (per 1-SD increase)	0.70 (0.62–0.80)	0.79 (0.68–0.91)	0.87 (0.74–1.02)
PEF quartiles Q1			
(ref)	1.00	1.00	1.00
Q2	0.75 (0.55–1.01)	0.82 (0.61–1.12)	0.92 (0.67–1.26)
Q3	0.54 (0.38–0.75)	0.64 (0.45–0.90)	0.75 (0.52–1.08)
Q4	0.46 (0.32–0.65)	0.63 (0.43–0.93)	0.80 (0.53–1.20)
*P* for trend	*<*.001	.007	.157

Values are presented as hazard ratio (95% confidence interval).

ADL = activities of daily living, BMI = body mass index, CESD-10 = 10-item Center for Epidemiologic Studies Depression Scale, IADL = instrumental activities of daily living, PEF = peak expiratory flow, SD = standard deviation.

* Unadjusted.

† Adjusted for age, sex, hukou type, education level, marital status.

‡ Broader conservative/exploratory model, additionally adjusted for IADL, ADL, cognitive score, CESD-10, BMI, hypertension, diabetes, cancer, heart disease, lung disease, stroke, arthritis, physical disabilities, smoking, drinking, hospitalization in the past year, falls in the past year, stratified by self-rated health.

In quartile analyses, compared with Q1, the HR for Q4 was 0.46 (95% CI: 0.32–0.65) in the crude model and 0.63 (95% CI: 0.43–0.93) in model 2. In model 3, quartile-specific estimates were further attenuated (Q4 vs Q1: HR 0.80, 95% CI: 0.53–1.20; Q3 vs Q1: HR 0.75, 95% CI: 0.52–1.08). The *P* for trend across quartiles was < 0.001 (model 1), 0.007 (model 2), and 0.157 (model 3). This attenuation pattern occurred after broader adjustment for variables plausibly related to functional vulnerability and pathway-related domains, as specified in the prespecified conceptual framework (Fig. S1, Supplemental Digital Content 1).

### 3.4. Supplementary discrimination analyses

Supplementary discrimination analyses showed progressive improvement in discrimination from model 1 to model 3 (Table S2, Supplemental Digital Content 2). The C-index was 0.594 for model 1, 0.616 for model 2, and 0.685 for model 3. Corresponding time-dependent AUCs at 2 years were 0.584, 0.599, and 0.688; at 5 years, 0.588, 0.606, and 0.688; and at 7 years, 0.601, 0.628, and 0.694, respectively. These findings indicate limited discrimination for the PEF-only and primary confounder-adjusted models and better model-level discrimination for the broader model incorporating health, functional, and comorbidity-related variables.

### 3.5. Kaplan–Meier curves

Kaplan–Meier curves demonstrated separation in hip fracture–free survival across baseline PEF quartiles over follow-up (Fig. 2). The overall difference among quartiles was statistically significant (log-rank *P* < .001). Using Q1 as the reference, pooled pairwise comparisons indicated lower risk in Q3 (pooled HR 0.54, 95% CI: 0.38–0.75; *P* < .001) and Q4 (pooled HR 0.46, 95% CI: 0.32–0.65; *P* < .001), while the comparison for Q2 was borderline (pooled HR 0.75, 95% CI: 0.55–1.01; *P* = .056).

**Figure 2.**
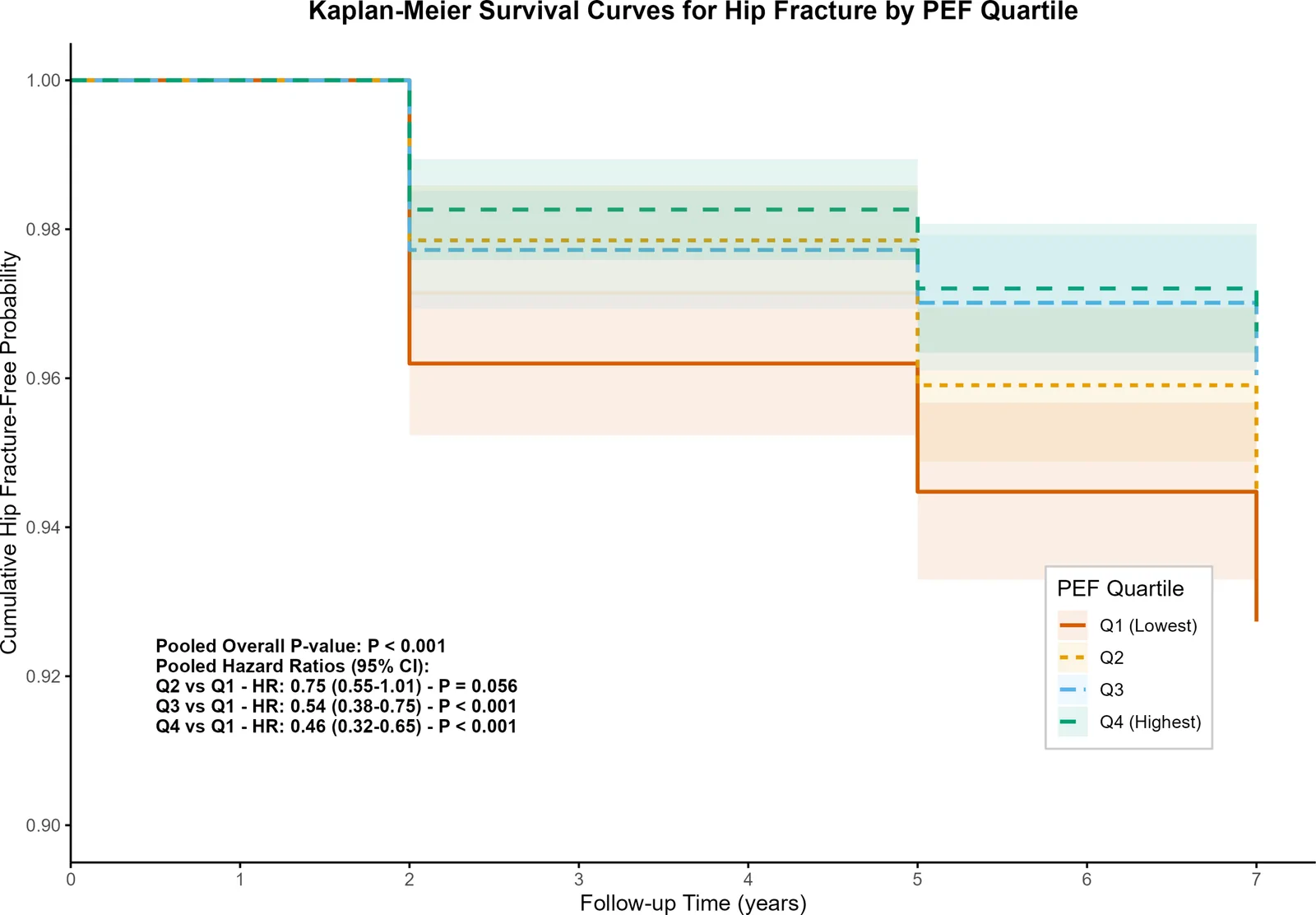
Kaplan–Meier curves for incident hip fracture by baseline peak expiratory flow quartiles. Kaplan–Meier estimates of hip fracture-free survival across quartiles of baseline PEF (Q1 lowest to Q4 highest). Shaded bands indicate 95% confidence intervals. Hazard ratios for Q2–Q4 versus Q1 were obtained from Cox models fitted within each imputed dataset and pooled across imputations. CI = confidence interval, HR = hazard ratio, PEF = peak expiratory flow.

### 3.6. Dose–response analysis

RCS analyses showed an approximately linear inverse association between continuous PEF and hip fracture risk (Fig. 3). There was no evidence of nonlinearity (*P* for nonlinearity = .866).

**Figure 3.**
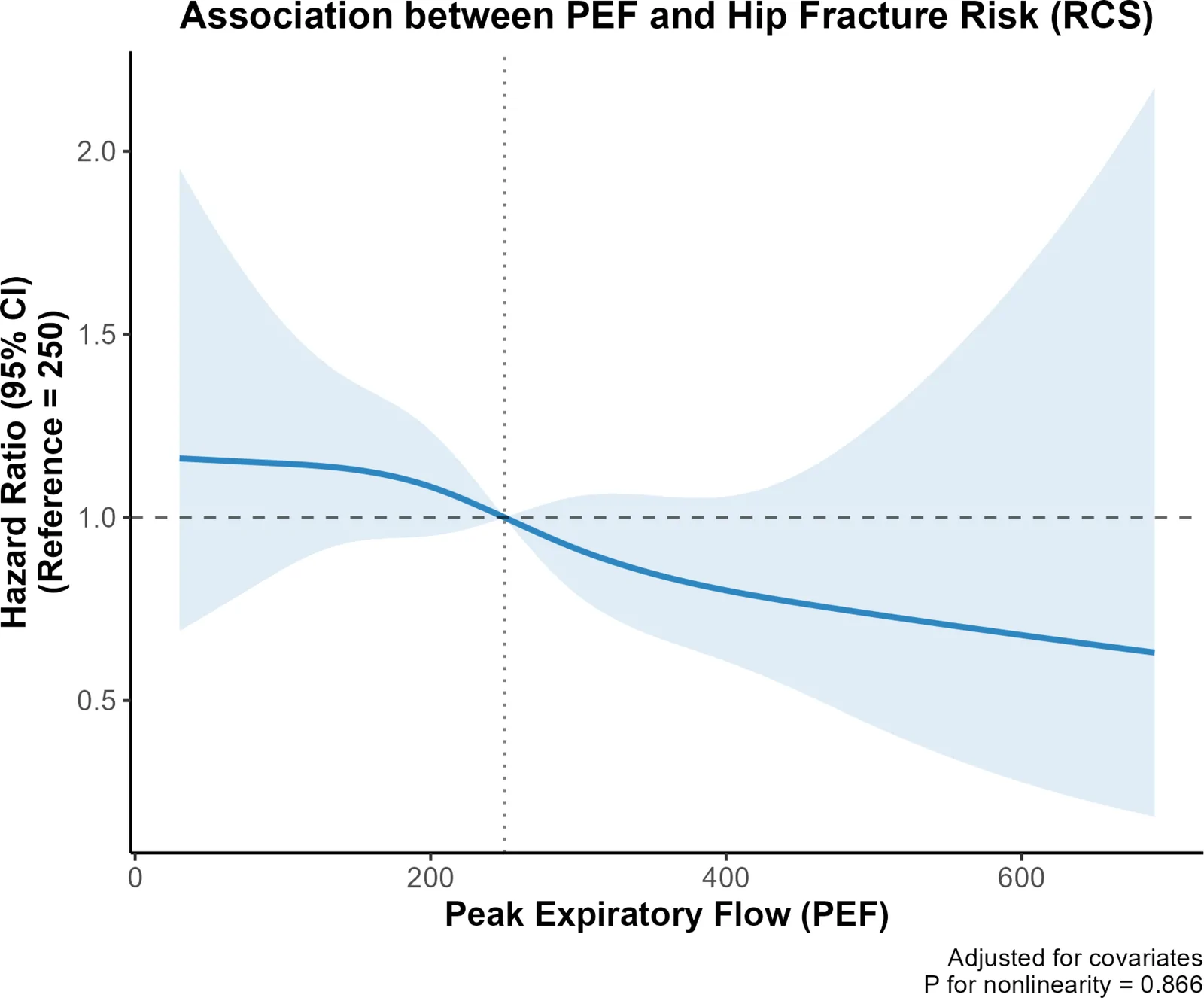
Restricted cubic spline analysis of baseline peak expiratory flow and incident hip fracture risk. Adjusted hazard ratios and 95% confidence intervals (shaded area) for incident hip fracture are shown across baseline PEF levels. The reference value was the median PEF. There was no evidence of nonlinearity (*P* for nonlinearity = .866). CI = confidence interval, PEF = peak expiratory flow, RCS = restricted cubic splines.

### 3.7. Secondary and sensitivity analyses

In pathway-adjusted models, the per 1-SD association between PEF and hip fracture remained below 1.0 when additionally adjusting for functional status (ADL/IADL; HR 0.85, 95% CI: 0.73–0.99), depressive symptoms and cognitive score (HR 0.86, 95% CI: 0.74–1.00), or falls (HR 0.84, 95% CI: 0.72–0.98), compared with the base estimate (HR 0.84, 95% CI: 0.72–0.97). After simultaneous adjustment for all pathway indicators, the estimate was further attenuated and not statistically significant (HR 0.87, 95% CI: 0.74–1.02). In quartile-based pathway models, point estimates for Q2–Q4 versus Q1 remained <1.0, with 95% CIs crossing 1.0 after additional pathway adjustment (Fig. 4).

**Figure 4.**
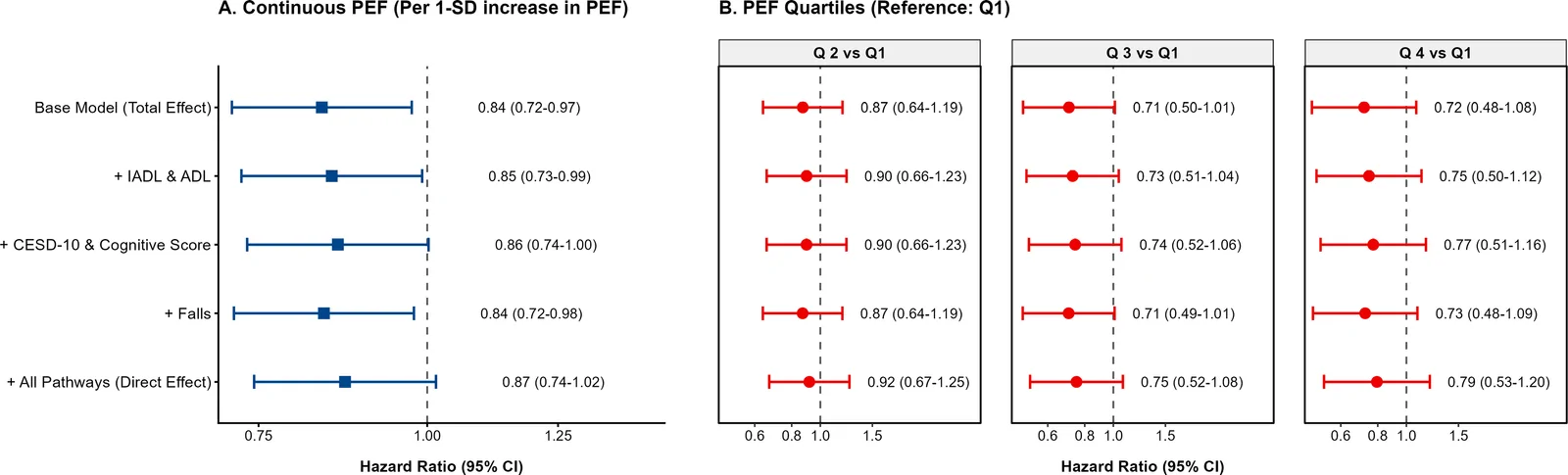
Sensitivity analyses for the association between baseline peak expiratory flow and incident hip fracture. HRs and 95% CIs are shown for incident hip fracture per 1-SD increase in baseline PEF (Panel A) and for PEF quartiles Q2–Q4 versus Q1 (Panel B) across sensitivity models. The base model included the same covariates as model 3 in Table 2 and was stratified by self-rated health, except that ADL, IADL, cognitive score, CESD-10, and falls in the past year were not included; subsequent models sequentially added these potential pathway-related variables, with the final model including all of them. Points indicate HRs and horizontal bars indicate 95% CIs. PEF, peak expiratory flow; ADL = activities of daily living, CESD-10 = 10-item Center for Epidemiologic Studies Depression Scale, CI = confidence interval, HR = hazard ratio, IADL = instrumental activities of daily living, PEF = peak expiratory flow, SD = standard deviation.

Discrete-time complementary log–log models produced estimates consistent in direction with the Cox analyses. Results were also directionally consistent in analyses excluding early events by the 2015 wave and excluding participants who died during follow-up (Fig. 5). Formal competing-risk regression for death was not feasible because sufficiently complete time-to-death information was unavailable.

**Figure 5.**
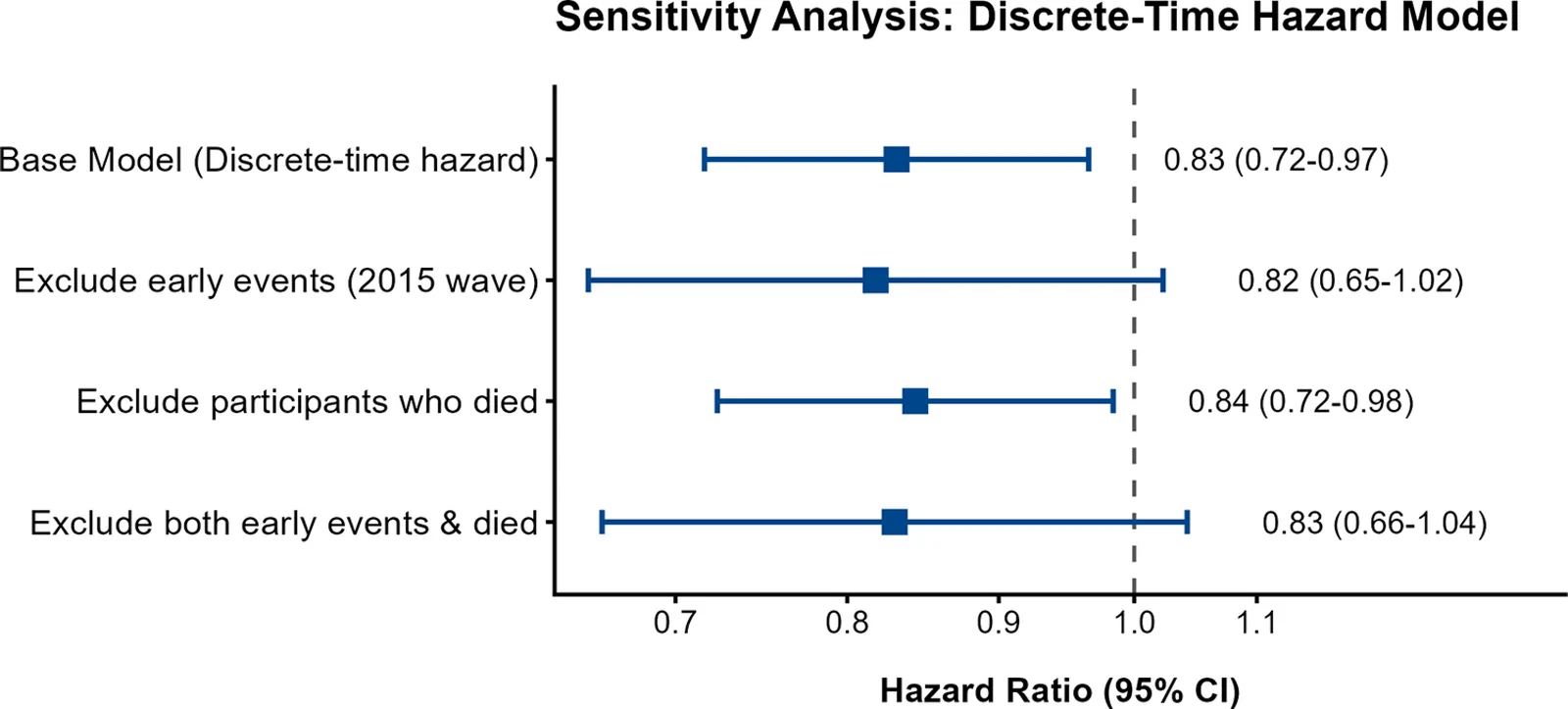
Discrete-time complementary log–log models and exclusion-based sensitivity analyses for baseline peak expiratory flow and incident hip fracture. Pooled hazard ratios and 95% confidence intervals for incident hip fracture per 1-SD increase in baseline PEF are shown for the discrete-time hazard model and for sensitivity analyses excluding early events, deaths during follow-up, or both. The base model was the same as in Figure 4. Points indicate hazard ratios and horizontal bars indicate 95% confidence intervals. PEF = peak expiratory flow, SD = standard deviation.

### 3.8. Subgroup and interaction analyses

In prespecified subgroup analyses (per 1-SD increase in PEF), effect estimates were generally directionally consistent across strata. Although some stratum-specific estimates differed numerically, no statistically significant interactions were detected for age, sex, education level, marital status, hukou type, hypertension, diabetes, history of falls, depressive symptoms, BMI, or smoking status (all *P* for interaction > 0.05). For BMI categories, HRs were: <18.5 kg/m^2^, 1.03 (95% CI: 0.53–2.02); 18.5–24 kg/m^2^, 0.85 (95% CI: 0.69–1.05); and >24 kg/m^2^, 0.74 (95% CI: 0.57–0.96). For smoking status, HRs were: never smokers, 0.74 (95% CI: 0.60–0.92); former smokers, 0.84 (95% CI: 0.55–1.29); and current smokers, 1.10 (95% CI: 0.77–1.57). Some subgroup estimates were imprecise due to limited numbers of events in specific strata (Fig. 6).

**Figure 6.**
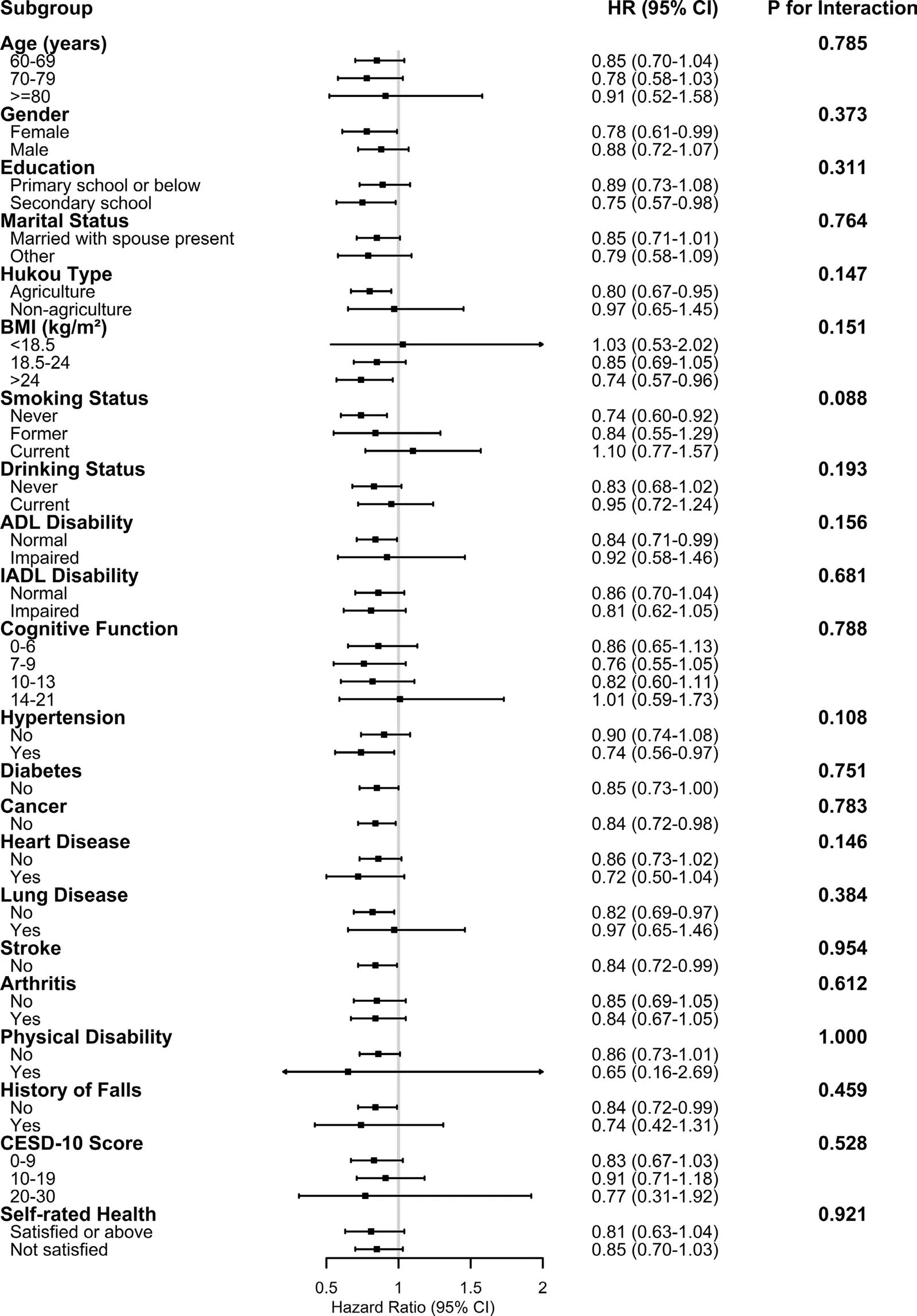
Subgroup analyses of baseline peak expiratory flow and incident hip fracture risk. Hazard ratios and 95% confidence intervals for incident hip fracture per 1-SD increase in baseline peak expiratory flow are shown across prespecified subgroups. Points indicate hazard ratios and horizontal bars indicate 95% confidence intervals. ADL = activities of daily living, BMI = body mass index, CESD-10 = 10-item Center for Epidemiologic Studies Depression Scale, CI = confidence interval, HR = hazard ratio, IADL = instrumental activities of daily living, SD = standard deviation.

## 4. Discussion

In this nationally representative prospective cohort of community-dwelling older Chinese adults from CHARLS, higher baseline PEF showed an inverse association with incident hip fracture in the crude and primary confounder-adjusted models, but this association was attenuated to non-significance after broader adjustment in model 3. Because several variables added in model 3 may plausibly lie along the pathway linking lower PEF to hip fracture risk, this attenuation should be interpreted cautiously. Accordingly, model 2 is best viewed as the primary confounder-adjusted model, whereas model 3 is more appropriately interpreted as a conservative/exploratory attenuation model rather than a strict causal model.

Our findings are consistent with prior evidence linking impaired pulmonary function and chronic respiratory disease with skeletal fragility and hip fracture risk.^[11,12,14]^ However, most previous studies focused on formal spirometric indices or clinically defined chronic obstructive pulmonary disease, whereas the present study evaluated PEF, a rapid and low-cost measure feasible for large-scale surveys and routine geriatric assessment. Because PEF is effort-dependent and influenced by participant cooperation, technique, and momentary condition, it should be interpreted as a pragmatic field measure of respiratory-related functional status rather than a substitute for comprehensive spirometric assessment.^[18]^

A key contribution of the present analysis is that the inverse PEF-hip fracture association weakened after broader adjustment, including variables closely aligned with frailty and intrinsic capacity, such as ADL/IADL, cognitive score, 10-item Center for Epidemiologic Studies Depression Scale, and falls. This attenuation is consistent with the interpretation that lower PEF may capture aspects of reduced physiologic reserve or broader vulnerability that overlap with established determinants of fracture risk. However, attenuation after broader adjustment cannot distinguish causal mediation from shared vulnerability pathways, residual confounding, or correlations among functional, cognitive, depressive, and fall-related domains. Therefore, these analyses should be interpreted as exploratory attenuation analyses rather than formal mediation analyses. Supporting this cautious interpretation, prior evidence links low PEF with injurious falls, sarcopenia, and respiratory muscle dysfunction, all of which may contribute to neuromuscular capacity, functional reserve, and fall susceptibility.^[9,13,21]^

Several factors may help explain the attenuation observed after broader adjustment. First, residual confounding cannot be excluded because the 2013 CHARLS data lacked directly comparable measures of physical activity, detailed nutritional status, and clinically informative medication use, such as corticosteroids or osteoporosis treatments.^[22,23]^ Second, PEF was effort-dependent and measured only once at baseline; therefore, non-differential measurement error and inability to capture longitudinal respiratory change may have diluted modest associations. Third, although the cohort was large, incident hip fractures were relatively infrequent, and extensive covariate adjustment may have increased model variance.

The supplementary discrimination analyses provide additional context. Discrimination was limited for the unadjusted PEF-only model and improved only modestly in the primary confounder-adjusted model, whereas the broader model 3 showed the highest C-index and time-dependent AUCs. This pattern suggests that overall risk stratification is improved more by incorporating broader information on health status, function, comorbidity burden, and falls than by PEF alone. These analyses therefore support the interpretation that PEF should not be viewed as a strong stand-alone fracture predictor but may contribute as 1 component of a broader risk profile. These discrimination analyses were supplementary and apparent, rather than externally validated predictive assessments, and should therefore be interpreted cautiously.

This study has several strengths. The prospective cohort design establishes the temporal sequence between baseline PEF and subsequent hip fracture; the analysis leveraged the nationally representative CHARLS cohort with standardized interviews and physical assessments^[16]^; follow-up extended to approximately 7 years; and we implemented a comprehensive analytic framework including Cox models with proportional hazards diagnostics, Kaplan–Meier analyses, RCS, prespecified subgroup assessments, and multiple sensitivity analyses. Importantly, we adjusted for a wide range of prespecified demographic, socioeconomic, lifestyle, functional, and comorbidity variables, which enabled a stringent evaluation of attenuation across models.

Several limitations should be noted. First, this was an observational analysis and cannot establish causality; residual confounding from unmeasured factors remains possible, particularly given unavailable data on physical activity, nutritional status, and clinically informative medication use. Second, incident hip fracture was ascertained by self-report rather than adjudicated medical records or imaging. Although previous validation studies in older epidemiologic cohorts suggest that self-reported fractures, particularly major fractures, can have acceptable validity for population-based research, some outcome misclassification remains possible.^[19,20]^ Such misclassification would more plausibly be non-differential with respect to baseline PEF and therefore bias associations toward the null. Third, PEF was measured at a single baseline time point and is inherently effort-dependent; thus, it may not fully capture longer-term respiratory trajectories and may be more variable than formal spirometric indices. Fourth, death is a clinically relevant competing event in older populations. However, because sufficiently complete time-to-death information was unavailable for most deceased participants, formal competing-risk regression was not feasible in this dataset, and some bias related to competing mortality cannot be excluded. In addition, survivor bias may have affected the observed association: participants with low PEF and high mortality risk may have died or been lost to follow-up before a hip fracture could be reported, which could have led to underestimation of the PEF–hip fracture association. Finally, because participants were older Chinese adults, extrapolation to other populations should be cautious.

From a clinical and public health perspective, our findings support a cautious reframing of PEF. Although lower PEF was associated with higher hip fracture risk in less-adjusted models, the association was substantially attenuated after broader adjustment, and discrimination analyses showed that PEF alone had limited predictive performance. Accordingly, PEF should not be interpreted as a stand-alone fracture risk predictor. Rather, low PEF may be viewed as a pragmatic clinical signal prompting broader geriatric assessment and closer attention to modifiable fall-, frailty-, and comorbidity-related domains.

## 5. Conclusion

In this prospective CHARLS cohort of community-dwelling older Chinese adults, higher baseline PEF was associated with lower incident hip fracture risk in crude and primary confounder-adjusted analyses, but the association was attenuated and no longer statistically significant after broader adjustment. These findings suggest that baseline PEF has limited stand-alone prognostic value for incident hip fracture and that the observed attenuation after broader adjustment is consistent with overlapping influences of functional, health status, and vulnerability-related domains. Low PEF may therefore be better interpreted as a pragmatic signal prompting broader geriatric assessment rather than as a stand-alone fracture risk marker.

## Acknowledgments

The authors thank the CHARLS team for providing high-quality, publicly available data. We also express our gratitude to all the participants of the CHARLS survey.

## Author contributions

**Conceptualization:** Wei Chen, Zongfeng Chen.

**Data curation:** Yan Wang, Wanfei Wu.

**Methodology:** Wei Chen, Zongfeng Chen.

**Project administration:** Yufeng Zhao, Jun Fei.

**Supervision:** Yufeng Zhao, Jun Fei.

**Validation:** Yan Wang, Wanfei Wu.

**Visualization:** Yan Wang, Wanfei Wu.

**Writing – original draft:** Wei Chen.

**Writing – review & editing:** Yufeng Zhao, Jun Fei.






