# Supplementary material for: Baseline peak expiratory flow and incident hip fracture in older Chinese adults: A prospective CHARLS cohort study
Source: Medicine (Baltimore). 2026 Oct 23;105(43):e51167. doi: 10.1097/MD.0000000000051167 (PMC13706453; doi:10.1097/MD.0000000000051167)
Supplement: Supplementary file 1 [file medi-105-e51167-s002.docx]

**Supplementary Materials**

# **Supplementary Table S2.**

# **Discrimination measures for Model 1, Model 2, and Model 3 across multiply imputed datasets**

| Model | C-index | AUC at 2 years | AUC at 5 years | AUC at 7 years |
| --- | --- | --- | --- | --- |
| Model 1 | 0.594 (0.000) | 0.584 (0.000) | 0.588 (0.000) | 0.601 (0.000) |
| Model 2 | 0.616 (0.000) | 0.599 (0.000) | 0.606 (0.000) | 0.628 (0.000) |
| Model 3 | 0.685 (0.004) | 0.688 (0.006) | 0.688 (0.005) | 0.694 (0.003) |

Values are presented as mean (SD) across 20 imputed datasets; SD reflects between-imputation variability.

Model 1: Unadjusted model with peak expiratory flow (PEF) only.

Model 2: Primary confounder-adjusted model, including PEF, age, sex, hukou type, education level, and marital status.

Model 3: Broader conservative/exploratory model, additionally including functional status, cognitive score, depressive symptoms, body mass index, baseline comorbidities, smoking, drinking, hospitalization in the past year, number of falls in the past year, and stratification by self-rated health.

AUC, area under the receiver operating characteristic curve; PEF, peak expiratory flow; SD, standard deviation.
